# IP_3_ 3-Kinase Opposes NGF Driven Neurite Outgrowth

**DOI:** 10.1371/journal.pone.0032386

**Published:** 2012-02-22

**Authors:** Richard Eva, Dalila Bouyoucef-Cherchalli, Kalpana Patel, Peter J. Cullen, George Banting

**Affiliations:** 1 Department of Biochemistry, School of Medical Sciences, University of Bristol, Bristol, United Kingdom; 2 The Henry Wellcome Integrated Signalling Laboratories, Department of Biochemistry, School of Medical Sciences, University of Bristol, Bristol, United Kingdom; 3 Department of High-Throughput Biology-Cell Physiology, GlaxoSmithKline, Harlow, United Kingdom; Cardiff University, United Kingdom

## Abstract

The inositol (1,4,5) trisphosphate 3-kinases comprise a family of enzymes (A, B, and C) that phosphorylate the calcium mobilising molecule inositol (1,4,5) trisphosphate (IP_3_) to generate inositol (1,3,4,5) tetrakisphosphate. This molecule can function as a second messenger, but its roles are not completely understood. The A isoform of inositol (1,4,5) trisphosphate 3-kinase localises to filamentous actin within dendritic spines in the hippocampus and is implicated in the regulation of spine morphology and long term potentiation, however the mechanisms through which it signals in neuronal cells are not completely understood. We have used NGF driven neurite outgrowth from PC12 cells as a platform to examine the impact of signaling via inositol (1,4,5) trisphosphate 3-kinase activity in a neuronal cell. We have found that the catalytic activity of the enzyme opposes neurite outgrowth, whilst pharmacological inhibition of inositol (1,4,5) trisphosphate 3-kinase leads to a significant increase in neurite outgrowth, and we show that the reduction in neurite outgrowth in response to inositol (1,4,5) trisphosphate 3-kinase activity correlates with reduced ERK activity as determined by western blotting using phosphorylation-specific antibodies. Our findings suggest a novel neuronal signaling pathway linking metabolism of IP_3_ to signaling via ERK.

## Introduction

Numerous ligand-operated signaling pathways involve the second-messenger inositol (1,4,5) trisphosphate (IP_3_), which is generated by the action of phospholipase C (PLC) on phopsphatidylinositol (4,5) bisphosphate (PIP_2_). Once generated, IP_3_ can either be metabolized by IP_3_ 5-phosphatases to generate the inactive inositol bisphosphate (IP_2_), or can be further phosphorylated by a family of IP_3_ 3-kinases, to generate inositol (1,3,4,5) tetrakisphosphate (IP_4_). IP_3_ is a second messenger which functions by binding to the IP_3_ receptor at the ER, resulting in the release of stored calcium [1]. IP_4_ is also recognized as a second messenger but its cellular functions are not fully understood [2], [3], [4]. There are three isoforms of IP_3_ 3-kinase. These have been designated A, B and C (IP_3_ 3-KA, IP_3_ 3-KB and IP_3_ 3-KC) [5]. IP_3_ 3-KB and IP_3_ 3-KC are expressed in most tissues, whilst IP_3_ 3-KA expression is enriched in the central nervous system [6], [7], [8]. IP_3_ 3-KB is the most studied of the three isoforms; it has been assigned roles in the regulation and development of various cells in the immune system, functioning principally via the production of IP_4_. Three studies have implicated IP_3_ 3-KB in the development of T-cells [9], [10], [11], and it has also been implicated in B-cell development, selection and activation [12], [13], [14], as well as the regulation of myelopoesis and neutrophil signaling [15], [16]. However, whilst these studies have been instrumental in defining a physiological role for IP_3_ 3-KB, the cellular roles of IP_4_ remain unclear and the mechanisms by which it signals require elucidation. Some studies suggest that IP_4_ production in T-cells is necessary for efficient activation of the ERK pathway [9], [10], whilst it is also necessary for the activation of PLC in order for T-cells to develop correctly [11]. Others suggest that, in B-cells, IP_4_ signals solely by inhibiting store operated calcium channels [13], [14], whilst it is also reported that IP_4_ negatively regulates PIP_2_ mediated activation of the GTPase activating protein Gap1IP4BP resulting in attenuated signaling via ERK [12]. To further complicate the issue, it has been reported that IP_4_ negatively regulates phosphatidylinositol (3,4,5)-trisphosphate (PIP_3_) signaling in neutrophils [15], [16]. It is therefore evident that IP_4_ (as generated by IP_3_ 3-kinase) has the ability to impact on numerous key signaling pathways depending on factors such as the cell type in which IP_4_ is produced. In the brain, IP_3_ 3-KA is enriched in dendritic spines in the CA1 region of the hippocampus [8], and is upregulated during spatial learning tasks [17]. It is also implicated in the regulation of long term potentiation (LTP) via the generation of IP_4_ [18], [19], and regulates dendritic spine morphology by functioning as a scaffold for Rac1 [20], and as an F-actin-bundling protein [21]. However the full consequences of its catalytic activity in neurons are not clear.

We chose to investigate the consequences of IP_3_ 3-KA expression in a neuroendocrine cell line during differentiation to a neuronal phenotype. PC12 cells respond to NGF stimulation by halting proliferation, extending neurites and adopting a neuronal phenotype. This differentiation process occurs as a result of the binding of NGF to the TrkA receptor, which activates a number of well characterized signaling pathways [22]. We therefore chose to use NGF driven neurite outgrowth as a platform to investigate the potential consequences of IP_3_ 3-KA activity in neuronal cells. We report that stable high expression of IP_3_ 3-KA dramatically inhibits neurite outgrowth from PC12 cells after NGF stimulation. We present evidence that this inhibition is most likely as a result of IP_4_ production, and occurs due to the subsequent attenuation of signaling via ERK.

## Materials and Methods

### Expression constructs

pEGFP-C3-IP_3_ 3-KA, containing the full length rat inositol 1,4,5-trisphosphate 3-kinase A coding sequence cloned into the HindIII and BamH1 restriction sites of pEGFP-C3 (Clontech) [8]. 66-459 pEGFP-C3-IP_3_ 3-KA, containing bp198–1353 of the rat inositol 1,4,5-trisphosphate 3-kinase A coding sequence ligated into the HindIII and BamH1 restriction sites of pEGFP-C3 [8]. D260A/K262A pEGFP-C3-IP_3_ 3-KA, containing the rat inositol 1,4,5-trisphosphate 3-kinase A coding sequence ligated into the HindIII and BamH1 restriction sites (as above), but with D260A and K262A point mutations [23]. All of these constructs were a gift from Dr. Michael Schell, Uniformed Services University of the Health Sciences, USA.

### PC12 cell culture

Low passage number PC12 cells were acquired from the European Collection of Cell Cultures (ECACC), and routinely grown in RPMI 1640 media (Sigma) supplemented with 10% batch tested French FBS (Biosera), 1% penicillin/streptomycin, and 2 mM L-glutamine on flasks coated with type IV collagen (Sigma), according to ECACC guidelines. PC12 cells were differentiated in the usual media supplemented with 100 ng/ml NGF (Sigma).

### Generation of stable PC12 cell lines expressing GFP tagged constructs

PC12 cells were transfected with plasmid DNA using lipofectamine 2000 (Invitrogen) on 90 mm petri dishes and grown in the usual media plus 400 µg/ml neomycin (G418, Invitrogen). In order to generate clonal lines, cultures were left in selective media until isolated colonies were visible. These were screened for GFP expression, and isolated using sterile cloning rings and expanded in selective culture media. Observations of stable cell lines were made using several clonal lines to ensure against artefacts of clonal expression. Individual clonal lines were then selected for FACS sorting.

### FACS sorting

Flow cytometric analysis and sorting were performed using a MoFlo Fluorescence Activated Cell Sorter (Cytomation), or the BD FACS Vantage SE system (BD Biosciences). Cells derived from a single clonal line were sorted at 488 nm by virtue of their GFP signal to enrich for the approximate 25% of the cell population expressing the highest levels of fluorescence, and the approximate 25% of the population expressing the lowest levels of fluorescence.

### Confocal Microscopy

Cell imaging was performed by confocal microscopy using a Leica TCS-NT confocal laser-scanning microscope attached to a Leica DM RBE upright epifluorescence microscope under a 63× oil immersion objective. Fluorophores were excited at 488 nm and 568 nm using a krypton/argon laser. Images shown are maximum projections of stacks of confocal slices obtained throughout the entire depth of neurites. Images were processed using Adobe Photoshop 6.0 and Adobe Illustrator 10 (2).

### Quantitative Analysis of Neurite Outgrowth

Indices of neurite outgrowth were quantified using the Cellomics Arrayscan high content imaging system, using a 10× objective to acquire images of fluorescently labelled cells via automated microscopy. Images were then analysed using a dedicated proprietary algorithm (Cellomics). For experiments to determine the effects of stable expression on neurite outgrowth, cells were plated onto 96 well plates at a density of 2500 cells per well into 100 µl of the usual supplemented media containing 100 ng/ml NGF. Cells were cultured in the presence of NGF and fixed for analysis after four days. For the experiments to determine the effect of C5 inhibition of IP_3_ 3-kinase and XeC inhibition of the IP_3_ receptor, cells were plated in the presence of NGF and drug or vehicle (DMSO) on day one, and fixed on day four. All experiments were performed a minimum of three times. Cells were fixed with 4% paraformaldehyde containing 10% sucrose and 0.06% Hoescht 33342 dye (to label nuclei). Cells were then washed with a proprietary buffer (Cellomics), and labelled (by indirect immunofluorescence) with a proprietary antibody (Cellomics) to detect neurites, (Cellomics NOG HitKit). The Arrayscan was then used to capture images of Hoescht 33342-labelled nuclei, in order to identify individual cells, and then images were acquired of the fluorescently labelled neurites. Cellomics image analysis software (Neurite Outgrowth Algorithm) was then used to quantify neurite outgrowth. For this study these included the neurite outgrowth index and the average neurite length. The neurite outgrowth index is a measure of neurite bearing cells, and is defined as the percentage of neurons whose summed lengths are greater than a set threshold. This was set at 10 µm longer than the average width of a cell body, which was set at 15 µm. The origin of neurites was determined by the Hoescht label.

The average neurite length was calculated as the sum of neurite lengths from cells with outgrowth divided by the number of cells with outgrowth. Neurites and cell bodies were identified using the Cellomics Neurite Outgrowth HitKit, which uses a proprietary neuronal marker as a primary antibody, visualised with a secondary antibody conjugated to Alexa 488.

### Analysis of ERK activity

For experiments to determine ERK activity, serum starved PC12 cells were stimulated with 1 ng/ml NGF and lysed after various time points. ERK activity was then determined by western blot. A rabbit polyclonal antibody recognising p44/42 MAP Kinase (Cell Signalling Technology) was used to detect total ERK, whilst a rabbit polyclonal antibody recognising phospho-p44/42 MAP Kinase (Thr202/Tyr204) was used to detect Phosphorylated ERK. The phospho-specific antibody detects rat p42 and p44 MAP kinase (ERK2 and ERK1) only when phosphorylated at Thr202 and Tyr204 (Cell Signalling Technology). Primary antibody was detected using a horseradish peroxidase conjugated secondary antibody (DAKO) and visualised by enhanced chemiluminescence.

### Pharmacological agents

The following small molecule inhibitors were used: Inositol 1,4,5-trisphosphate 3-Kinase inhibitor referred to as “C5”, N2-(m-Trifluorobenzyl), N6-(p-nitrobenzyl) purine (Calbiochem/Merck) [24]. Xestospongin C, an inhibitor of IP_3_-mediated calcium release (Calbiochem/Merck) [25].

### Statistical Analysis

Data presented represent means of three experiments +/− SEM. Statistical analysis was performed using one-way ANOVA with *post-hoc* analysis, or by two-way ANOVA. Data were analysed using GraphPad Prism.

## Results

### Expression of IP_3_ 3-KA in PC12 Cells Results in an Inhibition of Neurite Outgrowth

**Figure 1:**
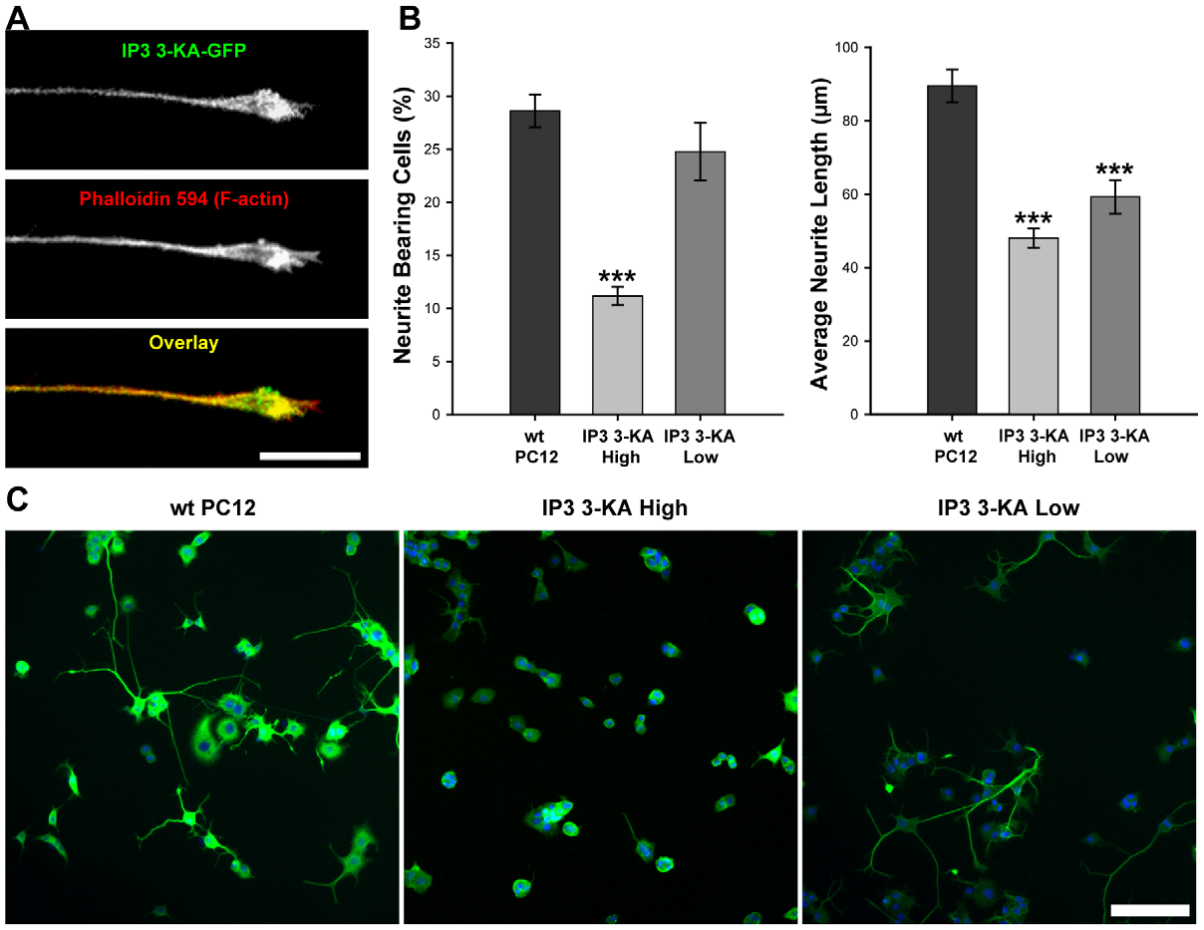
IP_3_ 3-KA co-localises with F-actin at the PC12 cell growth cone, and inhibits neurite outgrowth. (A) Growth cone of PC12 cell stably expressing high levels of IP_3_ 3-KA-GFP, fixed ten days after culture in the presence of 100 ng/ml NGF, and labeled with phalloidin-594. IP_3_ 3-KA-GFP co-localises with F-actin. Scale bar is 10 µm. (B) Quantitative analysis of the effects of IP_3_ 3-KA-GFP expression on neurite outgrowth. Neurite outgrowth was quantified using the Cellomics Arrayscan Neurite Outgrowth algorithm to measure the percentage of cells with neurites, and average neurite length (described in full in materials and methods). IP_3_ 3-KA expression results in an inhibition of neurite outgrowth. Data are presented as mean +/− SEM. ***p<0.001, by ANOVA with post-hoc analysis. (C) Arrayscan images of PC12 cells expressing varying levels of IP_3_ 3-KA-GFP, fixed four days after culture in the presence of 100 ng/ml NGF, and labeled using Hoescht 33342 and the Cellomics Neurite Outgrowth HitKit to identify neuritis. Scale bar is 100 µm.

In order to determine how IP_3_ 3-KA activity impacts on NGF driven neurite outgrowth from PC12 cells, we first generated numerous cell lines stably expressing GFP-tagged full length IP_3_ 3-KA. We sorted one of these lines by FACS to produce cell lines with different levels (designated high and low) of recombinant protein expression (as defined by the intensity of the GFP signal). Neuronal differentiation was induced by the addition of NGF, and we investigated the sub-cellular localisation of recombinant IP_3_ 3-KA-GFP by confocal microscopy. We initially observed that very few IP_3_ 3-KA-GFP expressing cells differentiated following the addition of NGF, and that the neurites of those cells that did differentiate were clearly shorter than their wild type counterparts. Where neurites were observed, IP_3_ 3-KA-GFP fluorescence was detected throughout their full length, and was enriched at the growth cone. We then used fluorescently labeled phalloidin to label F-actin, and confirmed that IP_3_ 3-KA-GFP colocalises with F-actin at the tip of growing neurites (Figure 1A).

The effects of IP_3_ 3-KA expression on neurite outgrowth were quantified using the Cellomics Arrayscan instrument for high content analysis and the Cellomics Neurite Outgrowth algorithm (detailed in the materials and methods section). Cells were incubated in the presence of NGF to induce neurite outgrowth and fixed after four days in culture. High levels of IP_3_ 3-KA expression resulted in a dramatic decrease (by 61%) in the number of cells with neurites compared with wild type control cells whilst lower levels of IP_3_ 3-KA expression resulted in less reduction (14%) (Figure 1B). We also analysed the average length of neurites and confirmed that expression of IP_3_ 3-KA resulted in cells with shorter neurites than those emanating from control cells (Figure 1B). Cells expressing high levels of IP_3_-3KA produced neurites that were on average 46% shorter than those produced by control cells, whilst cells expressing lower levels of IP_3_-3KA had neurites that were shorter on average by 34%. These data show a correlation between level of expression of IP_3_ 3-KA and phenotypic effect. They also demonstrate that IP_3_ 3-KA functions to oppose the actions of NGF on PC12 cells, and that this results in a dramatic reduction in neurite outgrowth, as can clearly be seen in images acquired using the Arrayscan (Figure 1C).

### The enzymatic activity of IP_3_ 3-KA is principally responsible for inhibiting NGF driven neurite outgrowth

**Figure 2:**
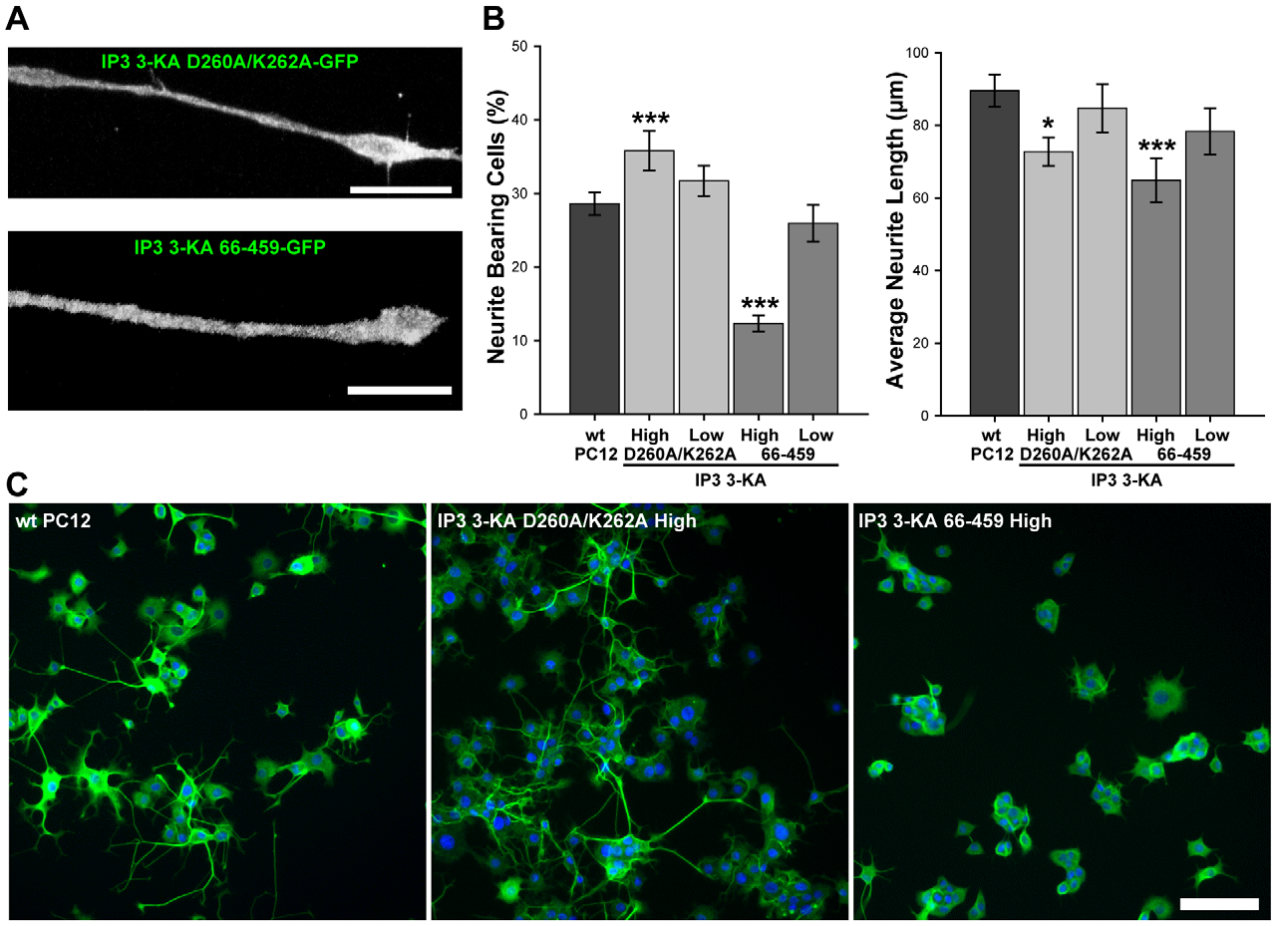
The catalytic activity of IP_3_ 3-KA opposes NGF-driven neurite outgrowth from PC12 cells. (A) Localisation of mutant IP_3_ 3-KA constructs in PC12 cell neurites. IP_3_ 3-KA D260A/K262A-GFP is enriched at the growth cone, whilst IP_3_ 3-KA 66-459-GFP is not. Scale bar is 10 µm. (B) Quantitative analysis of the effects of mutated IP_3_ 3-KA expression on neurite outgrowth. D260A/K262A-GFP overexpression results in an increase in the number of cells with neurites and decreases neurite length. IP_3_ 3-KA 66-459-GFP expression results in a decrease in both indices of neurite outgrowth. Data are presented as mean +/− SEM. *p<0.05, ***p<0.001, by ANOVA with post-hoc analysis. (C) Arrayscan images of PC12 cells expressing mutant IP_3_ 3-KA-GFP, fixed four days after culture in the presence of 100 ng/ml NGF, and labeled using Hoescht 33342 and the Cellomics Neurite Outgrowth HitKit to identify neuritis. Scale bar is 100 µm.

We reasoned that IP_3_ 3-KA could be inhibiting neurite outgrowth either by virtue of its catalytic activity, or by its ability to bind to F-actin. In order to address this issue, we generated cell lines stably expressing truncated or mutated IP_3_ 3-KA-GFP. 66-459 IP_3_ 3-KA-GFP lacks the N-terminal amino acids necessary for binding to F-actin and is therefore rendered cytosolic [8]. D260A/K262A IP_3_ 3-KA-GFP is identical to the full-length construct except for two point mutations in the catalytic domain which render the enzyme catalytically inactive [23], [26]. Cells were again sorted by FACS to produce high and low expressing stable cell lines. In contrast to cells expressing wild type IP_3_ 3-KA-GFP, cells expressing ‘kinase dead’ D260A/K262A IP_3_ 3-KA-GFP differentiated at a similar rate to their wild type counterparts in the presence of NGF. Kinase dead IP_3_ 3-KA-GFP localised in the same way as the wild type construct, and was enriched at the growth cone (Figure 2A, upper panel). Conversely, cytosolic 66-459 IP_3_ 3-KA-GFP was distributed uniformly along neurites, and was not enriched at the growth cone (Figure 2A lower panel). This suggests that the actin binding domain of IP_3_ 3-KA is required to enrich the enzyme at the growth cone of differentiating PC12 cells. We then quantified the effects of the mutant constructs on neurite outgrowth using the same criteria as for the full-length construct. High levels of kinase dead IP_3_ 3-KA-GFP expression resulted in an increase in neurite outgrowth of 25% in the number of cells with neurites compared with control cells (Figure 2B). Lower levels of expression had no effect. This suggests that the kinase dead construct is capable of acting as a partial dominant-negative. In contrast, high levels of expression of the cytosolic IP_3_ 3-KA-GFP construct resulted in a decrease (by 57%) in the number of cells with neurites compared with wild type controls (Figure 2B). These results suggest that the catalytic activity of the IP_3_ 3-KA is essential for the inhibition of differentiation caused by expression of the full-length enzyme. High levels of expression of the kinase dead IP_3_ 3-KA resulted in cells with shorter neurites (by 19%) compared with wild type controls (Figure 2B), whilst low levels of expression had no effect on neurite length. Cells expressing high levels of the cytosolic IP_3_ 3-KA exhibited neurites that were 28% shorter than controls (Figure 2B). This is not as marked an effect as that caused by expression of the full-length enzyme (p<0.001 by two-way ANOVA). Low levels of expression of the cytosolic IP_3_ 3-KA had no effect on neurite length, compared with a 32% reduction exhibited by the cells expressing low levels of the full-length enzyme. It would therefore appear that amino acids 1–65 are necessary to enrich IP_3_ 3-KA at growth cone actin, where it functions to inhibit neurite length, principally by virtue of its catalytic activity but also by regulating the actin cytoskeleton. Representative images of each of these cell lines (Figure 2C) highlight the fact that the catalytic activity of IP_3_ 3-KA is principally responsible for the inhibition of NGF-driven neurite outgrowth from PC12 cells.

### Inhibition of IP_3_ 3-kinase activity enhances neurite outgrowth from PC12 cells

**Figure 3:**
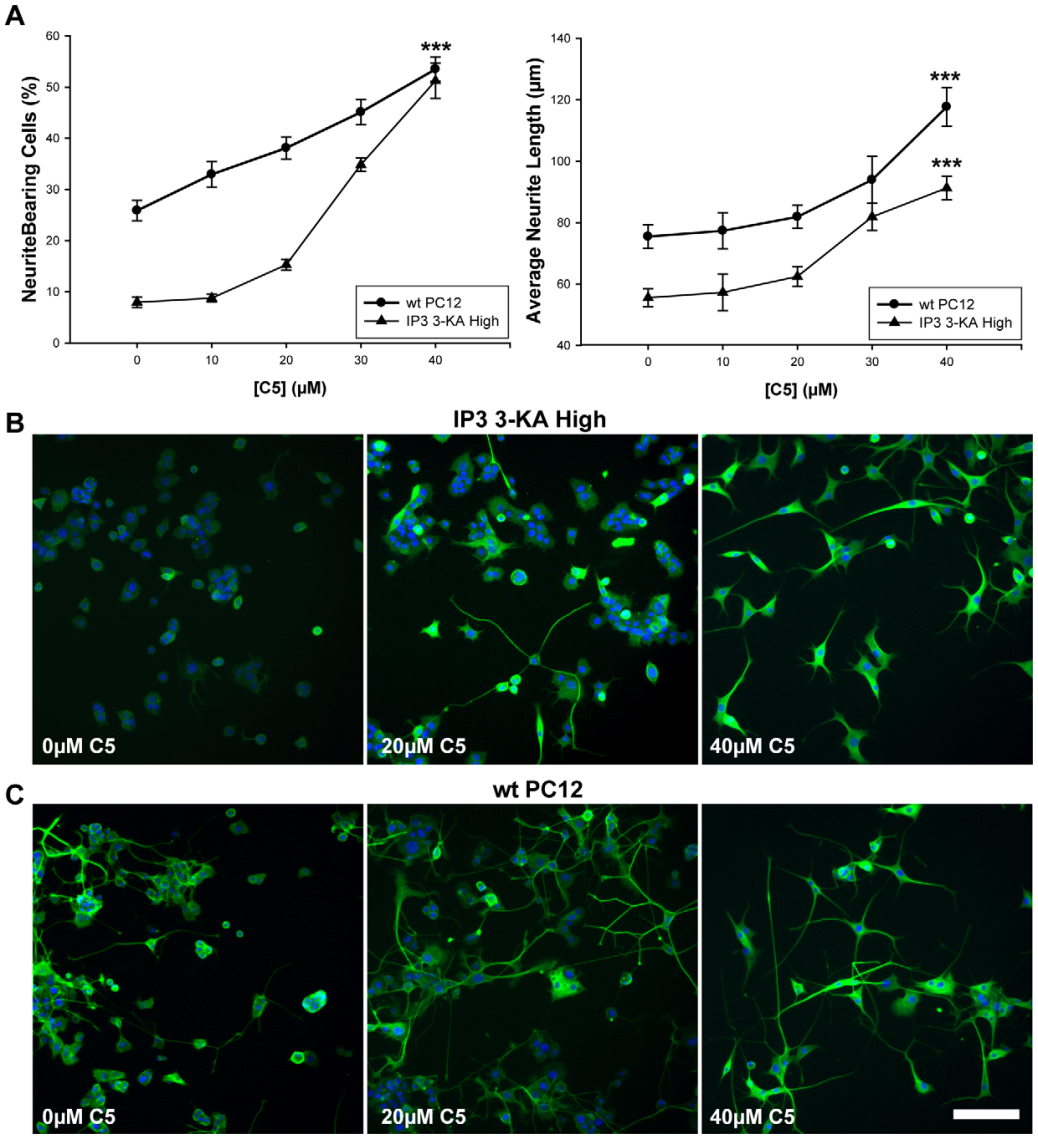
Inhibition of IP_3_ 3-kinase increases neurite outgrowth from PC12 cells, and IP_3_ 3-KA-PC12 cells. (A) Analysis of the effects of C5 on wild type PC12 cells and cells expressing high levels of IP_3_ 3-KA. Differentiating PC12 cells in the presence of C5 results in an increase in two indices of neurite outgrowth. Data are presented as mean +/− SEM. ***p<0.001, by ANOVA with post-hoc analysis. (B) Arrayscan images of PC12 cells expressing high levels of IP_3_ 3-KA, fixed four days after culture in the presence of 100 ng/ml NGF, and labeled using Hoescht 33342 and the Cellomics Neurite Outgrowth HitKit to identify neurites (using a 10× objective). (C) Arrayscan images of PC12 cells, fixed four days after culture in the presence of 100 ng/ml NGF, and labeled as for (B). Scale bar is 100 µm.

In order to investigate the effects of endogenous IP_3_ 3-kinase activity in PC12 cells, we investigated the effects of a number of reported IP_3_ 3-kinase inhibitors [24], [27] on neurite outgrowth. We found that most of these enhanced neurite outgrowth from IP_3_ 3-KA expressing cells, and chose molecule C5 for further investigation. C5 is a purine-based inhibitor of IP_3_ 3-kinase that is reported to have an IC50 of 10.2 µM *in vitro* [24]. We therefore investigated the effects of C5 on PC12 neurite outgrowth at concentrations of 10 to 40 µM. Cells expressing high levels of IP_3_ 3-KA were cultured in the presence of NGF and C5. Low doses (10–20 µM) of C5 had little effect. However at higher doses (30–40 µM) C5 overcame the inhibition on neurite outgrowth imposed by expression of IP_3_ 3-KA, giving rise to an increase in the number of cells with neurites, and an increase in average neurite length (Figure 3A). We then investigated the effects of IP_3_ 3-kinase inhibition on neurite outgrowth from wild type PC12 cells and found that increasing doses of C5 resulted in increases in both the number of cells with neurites and the length of neurites (Figure 3A). We found that the number of neurite bearing cells in wild type and IP_3_ 3-KA expressing cell types equalised as C5 dose increased, reflecting a dose dependent affinity of C5 for IP_3_ 3-kinase, and further indicating that the catalytic activity of IP_3_ 3-kinase opposes NGF driven PC12 cell differentiation. However, C5 inhibition did not completely overcome the inhibition of neurite length caused by IP_3_ 3-KA overexpression, again suggesting that it hinders neurite extension partly via its catalytic activity, but also by an additional mechanism (most likely by interfering with the actin cytoskeleton). Representative images of these cells underline the effects of IP3 3-kinase inhibition on neurite outgrowth from PC12 cells, illustrating that as the dose of C5 increases, neurites become increasingly more streamlined, and better defined (Figure 3B and C). These results demonstrate that endogenous IP_3_ 3-kinase activity opposes NGF driven neurite outgrowth from PC12 cells, and suggest that the enzyme functions to oppose the NGF dependant intracellular signals that trigger differentiation.

### IP_3_ 3-kinase activity attenuates ERK phosphorylation via a mechanism which appears distinct from the removal of IP_3_

**Figure 4:**
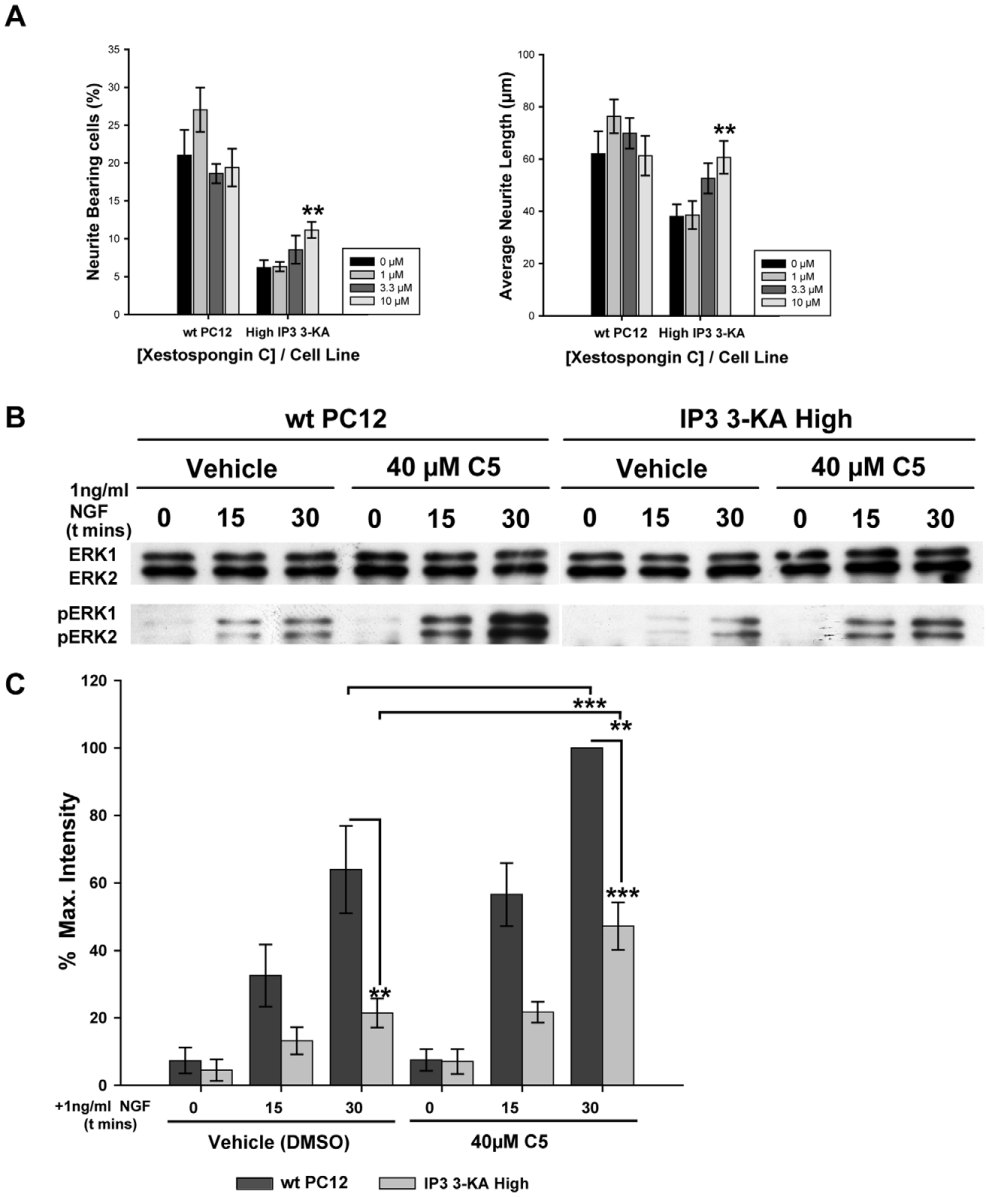
IP_3_ 3-KA activity results in attenuation of ERK phosphorylation. IP_3_ does not appear to be required for NGF driven neurite outgrowth. (A) Analysis of the effects of XeC blockade of the IP_3_ receptor, on PC12 cells expressing high levels of IP_3_ 3-KA and on wild type PC12 cells. XeC has no effect on control PC12 cells, but partially overcomes the inhibition of neurite outgrowth caused by IP_3_ 3-KA expression. Data are presented as mean +/− SEM. **p<0.01, by ANOVA with post-hoc analysis. (B) Determination of ERK activation in control PC12 cells and IP_3_ 3-KA expressing cells using a suboptimal dose of NGF, in the presence of either 40 µM C5 or vehicle. Lower blot represents ERK activation as detected by phospho-specific antibodies against ERK1 and ERK2. Upper blot represents total ERK, as detected using an antibody that detects both ERK1 and ERK2. All antibodies were visualised with an HRP conjugated secondary antibody. The blot shown is representative of three independent experiments. (C) Densitometric quantification of ERK activation, as depicted in (B). **p<0.01, Data are presented as mean +/− SEM. ***p<0.001, by ANOVA with post-hoc analysis. IP_3_ 3-KA expression attenuates ERK activation, whilst C5 inhibition of IP_3_ 3-kinase results in increased ERK activity.

We next questioned the mechanism by which IP_3_ 3-kinase activity opposes NGF driven neurite outgrowth, reasoning that the inhibition of neurite outgrowth caused by IP3KA over-expression was due either to the metabolism of IP_3_ or to increased production of IP_4_. We argued that if the increased metabolism of IP_3_ caused by IP_3_ 3-KA activity was responsible for inhibiting PC12 cell differentiation, then blockade of the IP_3_ receptor would have a similar effect. We therefore investigated the effects of IP_3_ receptor blockade during NGF driven neurite outgrowth using the cell permeable IP_3_ receptor inhibitor Xestospongin C (XeC). We found that IP_3_ receptor blockade with XeC had no effect on the number of wild type PC12 cells with neurites, or on neurite length (p<0.05 by ANOVA) (Figure 4A). This suggests that increased metabolism of IP_3_ caused by IP_3_ 3-KA overexpression is insufficient to inhibit PC12 cell differentiation. We also treated cells expressing high levels of IP_3_ 3-KA with NGF in the presence of XeC, and found that rather than compounding the effects of elevated IP_3_ 3-kinase activity, XeC overcame some of the IP_3_ 3-KA dependent inhibition of neurite outgrowth (Figure 4A). This suggests that reducing intracellular calcium by blocking its release from internal stores decreases IP_3_ 3-KA activity, and is in keeping with findings that IP_3_ 3-KA activity is calcium dependent [28], [29]. Our data are therefore consistent with the hypothesis that IP_3_ 3-kinase opposes the actions of NGF on PC12 cells via the generation of IP_4_, and are in agreement with previous findings that, whilst stimulation of PC12 cells with NGF results in the generation of IP_3_ and IP_4_, signaling via IP_3_ does not appear to contribute substantially to the differentiation process [30], [31]. Our data our also consistent with previous reports that XeC treatment has little effect on NGF-induced neurite outgrowth from PC12 cells [32], [33]. We therefore questioned the mechanisms by which IP_4_ might prevent PC12 cell differentiation, and focused on the ERK signaling pathway as sustained activation of ERKs is reported to be essential for the NGF mediated differentiation of PC12 cells [34].

We investigated the effects of C5 inhibition of IP_3_ 3-kinase on ERK phosphorylation in control cells and in cells expressing high levels of IP_3_ 3-KA. Serum starved cells were preincubated with either 40 µM C5 or vehicle for 30 minutes prior to stimulation with a low dose of NGF (1 ng/ml) and subsequently lysed at various time points. The onset of ERK phosphorylation was slow following stimulation with 1 ng/ml of NGF, and reached a peak after 30 minutes. Comparison of cells expressing high levels of IP_3_ 3-KA with wild-type controls revealed that ERK signaling is attenuated in the IP_3_ 3-KA expressing cells after stimulation with 1 ng/ml NGF (Figure 4 B and C). Furthermore, pre-incubation with 40 µM C5 resulted in an increase in the amount of phosphorylated ERKs in both the wild type cells, and cells expressing high levels of IP_3_ 3-KA. Quantification by densitometry revealed that 30 minutes after NGF stimulation, ERK phosphorylation is reduced by 66% in the cells expressing high levels of IP_3_ 3-KA compared with wild type controls (Figure 4C). Furthermore, inhibition of IP_3_ 3-kinase by C5 in wild type cells resulted in a 56% increase in ERK phosphorylation (Figure 4C). A similar effect was seen in cells expressing high levels of IP_3_ 3-KA. These results demonstrate that IP_3_ 3-kinase activity attenuates ERK signaling in NGF stimulated PC12 cells, contributing to an inhibition of neurite outgrowth, most likely as a result of IP_4_ production.

## Discussion

We have demonstrated that elevated expression of IP_3_ 3-KA dramatically inhibits NGF driven PC12 cell differentiation, and that this occurs principally as a result of the kinase activity of the enzyme. We have also shown that inhibiting endogenous IP_3_ 3-kinase leads to enhanced neurite outgrowth from NGF stimulated PC12 cells, suggesting that endogenous IP_3_ 3-kinase functions to oppose the actions of NGF on PC12 cells. This inhibition of PC12 cell differentiation involves the attenuation of signaling through ERK, and most likely occurs via the production of IP_4_ because inhibition of the IP_3_ receptor has no effect on neurite outgrowth from wild type PC12 cells. IP_3_ 3-kinase therefore appears to mediate a negative feedback mechanism which functions (via the production of IP_4_) to oppose the actions of NGF on PC12 cells.

We chose to investigate IP_3_ 3-KA in a model neuronal cell because it is this isoform of IP_3_ 3-kinase that is enriched in the dendritic spines of hippocampal neurons. The majority of previous studies examining the effects of signaling via IP_3_ 3-kinase have been undertaken in various immune cells (B-cells, T-cells and neutrophils [4], [9], [10], [11], [12], [13], [14], [15], [16], and there have been no studies to examine the impact of signaling via IP_3_ 3-kinase in neuronal cells. Whilst PC12 cells do not represent hippocampal neurons, they have provided a platform to investigate the consequences of signaling via IP3 3-kinase in a model system for neuronal differentiation. It is important to note that all of the elements of the pathway we have described are present in neurons in the hippocampus [35] where IP_3_ is generated post-synaptically by the activation of metabotropic glutamate receptors [36]. We have demonstrated, using a neuronal cell line, that IP_3_ 3-KA can act to attenuate ERK signaling and we suggest that if a similar process occurs within dendritic spines it would have significant consequences in terms of the regulation of post-synaptic excitability, and would be in keeping with the finding that mice lacking IP_3_ 3-KA have enhanced LTP [18]. Further studies are therefore required to determine how the pathway we have investigated in PC12 cells might be applied to the regulation of post-synaptic signaling.

We have not investigated the other signaling pathways activated by the binding of NGF to the TrkA receptor. TrkA also signals via phosphoinositide 3-kinase (PI 3-kinase) and PIP_3_, and IP_4_ is reported to inhibit PIP_3_ signaling since depletion of IP_4_ enhances PIP_3_ signaling in neutrophils [16]. Furthermore, IP_3_ 3-KB is reported to regulate myelopoiesis via the production of IP_4_ and the subsequent down-regulation of signaling via PIP_3_ and AKT [15]. It is possible then that increased IP_4_ production in PC12 cells expressing elevated levels of IP_3_ 3-KA could also inhibit signaling via PIP_3_, and contribute to the inhibition of neurite outgrowth. However, sustained ERK activation is considered to be the principal signal for NGF driven neurite outgrowth from PC12 cells, and it is possible that there is a direct link between IP_4_ production and ERK attenuation. The most likely candidates for this role are the IP_4_ binding proteins Gap1IP4BP and GAP1M. These are closely related proteins that are both reported to exhibit GTPase activating (GAP) activity towards Ras, whilst Gap1IP4BP is also reported to exhibit GAP activity towards the Ras related protein Rap1 [37]. As the sustained activation of ERKs responsible for NGF driven differentiation of PC12 cells is mediated by Rap1 [34], it may be that the dual GAP activity of Gap1IP4BP on both Ras and Rap is limiting the ERK dependent outgrowth process. In support of this hypothesis, it is reported that Gap1IP4BP is down-regulated during the differentiation of PC12 cells to a neuronal phenotype, suggesting that Gap1IP4BP exists as an obstacle to NGF driven neurite outgrowth [38]. It would therefore be interesting to determine whether changes in IP_4_ concentration (as a result of IP_3_ 3-kinase activity) affect GAP1IP4BP activity towards Rap1 and Ras in NGF stimulated PC12 cells. In summary, our findings suggest that IP_3_ 3-kinase functions to negatively regulate the NGF dependent signals that lead to the differentiation of PC12 cells. This most likely occurs via the production of IP_4_ leading to attenuation of signaling through ERK. We suggest that this signaling pathway may have relevance in other neuronal cells where it may be involved in the regulation of post synaptic signaling.
